# Reimagining Radiology: A Comprehensive Overview of Reviews at the Intersection of Mobile and Domiciliary Radiology over the Last Five Years

**DOI:** 10.3390/bioengineering11030216

**Published:** 2024-02-24

**Authors:** Graziano Lepri, Francesco Oddi, Rosario Alfio Gulino, Daniele Giansanti

**Affiliations:** 1 Azienda Unità Sanitaria Locale Umbria 1, Via Guerriero Guerra 21, 06127 Perugia, Italy; graziano.lepri@uslumbria1.it; 2 Facoltà di Ingegneria, Università di Tor Vergata, Via del Politecnico, 1, 00133 Roma, Italy; francesco.oddi@alumni.uniroma2.eu (F.O.); gulino@disp.uniroma2.it (R.A.G.); 3 Centro Nazionale TISP, Istituto Superiore di Sanità, Viale Regina Elena 299, 00161 Roma, Italy

**Keywords:** domiciliary radiology, domiciliary radiography, mobile radiology, mobile radiography

## Abstract

(Background) Domiciliary radiology, which originated in pioneering studies in 1958, has transformed healthcare, particularly during the COVID-19 pandemic, through advancements such as miniaturization and digitization. This evolution, driven by the synergy of advanced technologies and robust data networks, reshapes the intersection of domiciliary radiology and mobile technology in healthcare delivery. (Objective) The objective of this study is to overview the reviews in this field with reference to the last five years to face the state of development and integration of this practice in the health domain. (Methods) A review was conducted on PubMed and Scopus, applying a standard checklist and a qualification process. The outcome detected 21 studies. (Key Content and Findings) The exploration of mobile and domiciliary radiology unveils a compelling and optimistic perspective. Notable strides in this dynamic field include the integration of Artificial Intelligence (AI), revolutionary applications in telemedicine, and the educational potential of mobile devices. Post-COVID-19, telemedicine advances and the influential role of AI in pediatric radiology signify significant progress. Mobile mammography units emerge as a solution for underserved women, highlighting the crucial importance of early breast cancer detection. The investigation into domiciliary radiology, especially with mobile X-ray equipment, points toward a promising frontier, prompting in-depth research for comprehensive insights into its potential benefits for diverse populations. The study also identifies limitations and suggests future exploration in various domains of mobile and domiciliary radiology. A key recommendation stresses the strategic prioritization of multi-domain technology assessment initiatives, with scientific societies’ endorsement, emphasizing regulatory considerations for responsible and ethical technology integration in healthcare practices. The broader landscape of technology assessment should aim to be innovative, ethical, and aligned with societal needs and regulatory standards. (Conclusions) The dynamic state of the field is evident, with active exploration of new frontiers. This overview also provides a roadmap, urging scholars, industry players, and regulators to collectively contribute to the further integration of this technology in the health domain.

## 1. Introduction

### 1.1. Background

Embarking on a historical journey, the roots of domiciliary radiology trace back to an era preceding the digitalization wave and the establishment of standardized DICOM protocols. In 1958 [1], we registered the first pioneering studies starting the revolutionary path regarding domiciliary radiology, a time when mobile technology and its associated contributions were conspicuously absent [1]. Domiciliary radiology, since its inception, has emerged as a modern convenience and a profound game-changer. It liberates individuals from the anguish of arduous journeys and shields them from potential contagion in hospital corridors. The impact extends beyond patients, offering assistance to families burdened with travel logistics [2]. Venturing into the realm of remote execution reveals a complex tapestry involving a meticulous choreography of activities. Reshaping roles, navigating stringent radiological safety protocols, complying with regulations, deciphering evolving technologies, and considering non-technological resources like transportation contribute to the intricate dance of domiciliary radiology [2]. While this avant-garde approach lightens the load on hospital resources, it introduces a distinct set of challenges, demanding recalibration of workflows and strategic reallocation of attention and resources. The ongoing discourse about its merits and challenges is a testament to the dynamic equilibrium it seeks to establish. The true crucible of domiciliary radiology, including the pivotal role of mobile radiology units, emerged during the COVID-19 pandemic [3]. Crafted to cater to unique patient needs, this practice became an indispensable lifeline, especially for individuals with fragilities and disabilities. Its role in crisis management not only elevated visibility but also kindled newfound appreciation, underscoring resilience and adaptability in adversity. Once a niche chapter, the narrative of domiciliary radiology now stands tall as a testament to transformative healthcare practices in the 21st century. The intersection of advancements in specific technologies, Information and Communication Technology (ICT), and mobile technology has collectively made a significant impact, marking a transformative period in this field [4,5,6,7]. The evolution of domiciliary radiology, driven by continuous innovation, is marked by the miniaturization of technology and the relentless progress of digitization. Since the 1960s, there has been a transformative shift in healthcare and radiology, with specific technologies becoming key drivers. Miniaturization has revolutionized the practicality and portability of radiological instruments, enabling healthcare professionals to extend services beyond traditional settings [4,5]. The digital revolution has played a pivotal role, enhancing the efficiency and accessibility of radiological data. The seamless transition from analog to digital imaging has improved precision and paved the way for integrating cutting-edge technologies. This digital shift, coupled with advancements in data networks, has ushered in a new era of domiciliary radiology. The interconnected world, characterized by fast and stable data networks, empowers healthcare providers to conduct remote radiological procedures with unprecedented ease. The synergy between advanced technologies and robust data networks has not only fostered the growth of home-based and mobile radiology but also amplified capabilities, resulting in a paradigm shift in healthcare delivery [5]. Diagnostic services can now be seamlessly extended to the comfort of the patient’s home and to the mobile radiology interconnection. The journey from bulky technologies of the past to sleek, portable, and digitally empowered tools signifies a revolution in domiciliary radiology [6]. This evolution reflects a commitment to technological excellence and emphasizes a dedication to enhancing patient care through innovation and accessibility. The future of the intersection of domiciliary radiology and mobile technology following the COVID-19 pandemic appears poised for even greater strides, promising a continuum of advancements that will further redefine the healthcare delivery landscape [7].

### 1.2. Issues Emerging on the Horizon

In the evolving intersection of domiciliary and mobile radiology, a range of inquiries has propelled research into uncharted territories [2]. Artificial Intelligence and machine learning play a significant role in enhancing diagnostic precision. Simultaneously, the regulatory framework undergoes scrutiny alongside technological progress, exploring the intricate relationship between policies and the adoption of remote imaging. Patient outcomes, satisfaction, and the experiences of other involved actors become focal points, investigating the impact of remote imaging on well-being. Cybersecurity measures guarding data and infrastructure are crucial considerations. Economic aspects, including cost-effectiveness and accessibility, feature prominently in the discourse. Interdisciplinary collaboration optimizes domiciliary radiology services, emphasizing its impact on patient care. The roles of domiciliary radiology and mobile radiology in chronic disease management are explored, and attention shifts to challenges hindering adoption, with researchers seeking innovative solutions. Ethical considerations delicately address privacy and technology use, while patient empowerment and education emerge as vital components [6]. Together, these questions guide domiciliary radiology research, delving into technological advancements and broader human-centric considerations, shaping the dynamic healthcare landscape [7].

### 1.3. Defining the Grounds for Conducting a Review

The preceding introductory discussion not only raises crucial questions but also sheds light on the underlying reasons necessitating a thorough exploration of the developments and emerging issues surrounding this field of study of radiology. This need becomes particularly apparent in the wake of the technological revolution that unfolded in the second millennium, marked by miniaturization, digitization, and advancements in Information and Communication Technology (ICT), including the increasingly robust and stable capabilities of mobile connections. The overarching aim of this study is to conduct an exhaustive narrative review of existing reviews within the realm of the intersection of domiciliary radiology and mobile radiology over the past five years, including, therefore, also the advancements driven by the research during the COVID-19 pandemic. This comprehensive analysis seeks to delve into the multifaceted landscape of home-based radiology, considering technological advancements, evolving practices, and the broader implications for patient care.

#### Sub-Objectives Include the Following:

- Technological evolution: Examine the progression of technological innovations, such as miniaturization, digitization, and ICT, and their impact in this field related to radiology practices.
- Patient-centric implications: Explore the implications on patient experiences, outcomes, and overall satisfaction, with a focus on advancements in mobile connectivity.
- Interdisciplinary collaboration: Investigate the role of interdisciplinary collaboration in optimizing radiology services, considering the integration of various healthcare disciplines.
- Regulatory and ethical considerations: Analyze the regulatory and ethical considerations surrounding domiciliary and mobile radiology, particularly in the context of evolving technologies and practices.
- Comparative effectiveness: Assess the comparative effectiveness of mobile and domiciliary radiology against traditional in-hospital radiological procedures, considering factors like cost-effectiveness and diagnostic accuracy.

## 2. Methods

The narrative review of reviews used the ANDJ standardized checklist designed for narrative reviews [8]. The PubMed and Scopus databases were inserted in the overview. A qualification methodology was used to choose the studies based on the assessment of qualified parameters [9]. Based on [9], we evaluated each contribution based on key parameters:

- *N*1: *Clarity of study rationale in the introduction.*
- *N*2: *Appropriateness of work’s design.*
- *N*3: *Clarity in describing methods.*
- *N*4: *Clear presentation of results.*
- *N*5: *Justification and alignment of conclusions with results.*
- *N*6: *Adequate disclosure of conflicts of interest by authors.*

We assigned a graded score (1 to 5) to *N*1–N5.

For *N*6, we provided a binary assessment (Yes/No) for disclosure of conflicts.

We preselect studies meeting the following criteria:

*N*6 must be “Yes” for conflict disclosure.

The cumulative score for *N*1–*N*5 must exceed 3.

Only peer-reviewed studies were considered (including congress proceedings if peer-reviewed). The defined search query * was the following: =

*(mobile radiology[Title/Abstract]) OR (mobile radiography[Title/Abstract]) OR (domiciliary radiology[Title/Abstract]) OR (domiciliary radiography[Title/Abstract]) Filters: Review, Systematic Review, in the last 5 years Sort by: Most Recent*

The procedure-based overview identified 21 reviews, all matching 100% of the PubMed query [10] and 94% of the Scopus query.

*Preliminary trials included the composite key at the second row of Box 1. Nevertheless, our observations revealed that the outcomes were primarily associated with distinct areas, such as radiologists operating remotely from home (especially during the COVID-19 pandemic) and the expansive realm of teleradiology. These aspects, while noteworthy, fall outside the specific scope of this overview and warrant a more specialized discussion.

## 3. Results

The results from the overview of the reviews are arranged into two paragraphs: Section 3.1 reports a detailed analysis, while Section 3.2 reports the key findings (supported by a summary table (Table 1).

Of the 21 selected review studies [11,12,13,14,15,16,17,18,19,20,21,22,23,24,25,26,27,28,29,30,31], a total of 14 studies specifically focused on the themes of the review [13,14,15,16,17,18,20,21,23,24,26,27,28,31], while the remaining [11,12,19,22,25,29,30] had a focus on different topics.

**Table 1:** Key elements/points emerging from the overview of these studies with the focus.

Reference	Focus	Key Points
[13]	*Musculoskeletal Ultrasound*	- *Radiation-free and dynamic imaging tool*
		- *Addresses increasing demand for training opportunities*
[14]	*Mobile Devices Integration in the Health Domain*	- *Challenges in the widespread adoption of mobile devices*
		- *Call for a universal mobile application in healthcare*
[15]	*Telemedicine and COVID-19 Impact*	- *Transformative impact on healthcare organizations*
		- *Evolution of telemedicine areas, complementing traditional medicine*
[16]	*AI Applications in Pediatric Radiology*	- *Dominance of deep convolutional neural networks*
		- *Necessity for further exploration of AI’s value in optimizing doses*
[17]	*Mobile Mammography for Breast Cancer Screening*	- *Addresses disparities in breast cancer outcomes*
		- *Potential for earlier detection, especially in underserved women*
[18]	*Simulation-Based Training (SBT)*	- *Common use in allied health professions*
		- *Unclear impact on sustained skill enhancement, calls for further investigation*
[20]	*Focus on Diagnostic Radiology Standards in Low-Resource Settings*	- *Emphasis on the critical role of diagnostic imaging*
		- *Lack of evidence on standard quality control for mobile health units (MHUs)*
[21]	*Augmented Reality in Anatomy Education*	- *Identification and assessment of various AR modalities*
		- *Need for sufficiently powered studies and validated assessment tools*
[23]	*Domiciliary Radiology with Mobile X-ray Equipment*	- *Exploration of mobile X-ray equipment outside the hospital*
		- *Need for further research, considering limitations in language, databases, and grey literature*
[24]	*Trends in Ultrasound Imaging*	- *Emphasis on widespread use and portability of mobile devices*
		- *Developments in miniaturization and improved imaging performance*
[26]	*Quality Improvement Interventions in Radiology*	- *Improvements in operational efficiency, report turnaround time, and teamwork*
		- *Positive outcomes associated with the introduction of mobile radiography*
[27]	*Integration of Internet-Based Technology in Radiology*	- *Integral connection between radiology and the Internet*
		- *Seamless integration of Internet-based applications, including mobile devices and wearables*
[28]	*Mobile Diagnostic Imaging Device*	- *Review of Mobile MIM diagnostic imaging device on iOS mobile devices*
		- *Emphasis on features and usability, especially in radiation treatment plans and medical image analysis*
[31]	*Workflow Optimization with Mobile Tools*	- *Addressing challenges in communication and workflow efficiency*
		- *Development of a mobile tool to optimize workflow efficiency in academic radiology departments*

### 3.1. In-Depth Analysis of the Detected Reviews: A Comprehensive Overview

In Neubauer et al.’s research [13], the focus is on musculoskeletal ultrasound as a radiation-free and dynamic imaging tool that enhances diagnostic and therapeutic safety. The study aims to map the current state of musculoskeletal ultrasonography education, highlighting the increasing demand for training opportunities. The systematic literature search conducted in January 2022 reveals a diverse range of course concepts and programs in various disciplines, with a particular emphasis on residents in rheumatology, radiology, and physical medicine and rehabilitation. International institutions have proposed guidelines and curricula to standardize ultrasound training. The important role of the mobile devices is recognized. The study concludes that standardized musculoskeletal ultrasound curricula would enhance training and facilitate the implementation of new programs.

Kufel et al. [14] explore the utilization of mobile devices in medicine, emphasizing the challenges in their widespread adoption. The study compares the usability of mobile applications for diagnostic image evaluation with stationary descriptive stations. Despite differences in procedures, device availability, and regulatory frameworks, the research identifies both positive and negative features of portable methods for analyzing radiological images. The authors stress the need for a universal mobile application with convenient and simple usage in hospital infrastructure. Future research will focus on advancements in using mobile devices and applications in the medical sector.

Perrone et al.’s study [15] delves into the impact of the COVID-19 pandemic on healthcare organizations and the evolution of telemedicine areas. The review highlights the role of electronic health records, teleradiology (including mobile/domiciliary radiology), telecardiology, teledermatology, and other telemedicine applications in managing the challenges posed by the pandemic. The authors stress the importance of telemedicine as complementary to traditional medicine and discuss its significant applications in radiology and dermatology.

Ng et al. [16] address the crucial aspect of radiation dose optimization in pediatric radiology. The systematic review focuses on Artificial Intelligence (AI) techniques and architectures for dose optimization, highlighting the dominance of deep convolutional neural networks in reducing radiation dose without compromising diagnostic information. Mobile radiography devices were included in the study. The study emphasizes the necessity for further explorations of AI’s value in dose optimization for various modalities in pediatric radiology.

Trivedi et al. [17] examine the disparities in breast cancer outcomes among different populations and highlight the role of mobile mammography units in providing convenient screening services. The review discusses the history and benefits of mobile mammography, especially for underserved women, emphasizing its potential to enable earlier detection of breast cancer and alleviate the impact of missed screenings during the COVID-19 pandemic.

Heuer et al. [18] conducted a systematic review of the utilization of simulation-based training (SBT) in allied health professions. The study identifies the common use of SBT in paramedics, emergency medical technicians, and respiratory therapists. Almost half of the studies were conducted in a stationary or mobile simulation lab. While SBT proves effective in enhancing short-term measures like post-training skill improvement and participant confidence, its impact on sustained skill enhancement and patient outcomes remains unclear.

Dinar et al. [20] focus on diagnostic radiology standards in low-resource settings, conducting a systematic review of the quality control for mobile health units (MHUs). The study notes a lack of evidence on standard quality control for MHUs and emphasizes the critical role of diagnostic imaging in ensuring successful patient management in crisis situations. The authors call for investigations to assess the feasibility of different quality control standards in MHUs.

McBain et al. [21] conducted a scoping review to identify augmented reality (AR) modalities used in anatomy education and examined assessment tools for their performance. Four AR modalities were identified, including head-mounted display, projection, instrument and screen, and mobile devices. The assessment focused on usability, feasibility, and acceptability, with a recent interest in visuospatial ability, cognitive load, time on task, and academic achievement outcomes. The authors emphasized the need for sufficiently powered studies using validated assessment tools to better understand AR’s role in anatomical education.

Toppemberg et al. [23] center their scoping review on domiciliary radiology, exploring the use of mobile X-ray equipment outside the hospital. The review included 12 studies published between 2009 and 2020 and highlighted the potential benefits for various populations, such as hospice patients, those with intellectual disabilities, and psychiatric patients. The results suggested improvements in population health, image quality, and potential cost-effectiveness. However, the authors noted limitations in language, databases, and grey literature, emphasizing the need for further research.

Wang et al. [24] provide a comprehensive review of current trends in ultrasound imaging, emphasizing its widespread use, portability of mobile devices, and recent advancements. The review highlights developments in ultrasound systems, including their miniaturization, improved imaging performance, and lower costs. The authors discuss ultrasound’s expanded applications, such as molecular imaging, super-resolution imaging, and focused treatments with intravascular microbubbles. The article projects a promising future for ultrasound in solving various medical challenges.

Jabin et al. [26] conducted a review of quality improvement interventions in radiology, focusing on both staff and patient perspectives. The study includes 18 selected studies covering interventions such as health information technology, training and education, immediate and critical reporting, safety programs, and the introduction of mobile radiography. The results demonstrate improvements in operational efficiency, reported turnaround time, teamwork, communication, and patient safety. The introduction of mobile radiography demonstrated improvements in outcomes, such as improved operational and workflow efficiency, report turnaround time, and teamwork and communication.

The authors called for further research to explore additional dimensions of quality, cost, and risk versus benefit.

Gupta et al. [27] discuss the integral connection between radiology and the Internet, emphasizing the rapid rise of Internet-based technology in healthcare. The article highlighted the seamless integration of Internet-based applications in radiology, including the use of mobile devices and wearables. The authors explore the impact of the Internet of Things (IoT) on radiology workflows, resident and medical student education, research, and patient engagement.

Adusumilli et al. [28] reviewed the Mobile MIM diagnostic imaging device, which is available on the Apple App Store. The FDA-approved app allows physicians to visualize scans from multiple modalities on iOS mobile devices, focusing on radiation treatment plans and medical image analysis. The review discusses the app’s features and usability, emphasizing its strengths and limitations, including incompatibility with mammography.

Makary et al. [31] address the challenges of communication and workflow efficiency in radiology operations. They shared their experience developing a mobile tool to optimize workflow efficiency and streamline access to relevant information in academic radiology departments. The mobile tool aims to enhance communication among care team members, provide access to guidelines and schedules, and keep users informed about local and international news.

### 3.2. Common Findings and Key Findings

From these studies, we can identify more general common findings and more specific emerging themes.

The analyzed reviews cover a diverse range of topics related to mobile/domiciliary radiology, including the use of AR in anatomy education, [21] domiciliary radiology with mobile X-ray equipment [23], advancements in ultrasound imaging [24], quality improvement interventions [26], the integration of Internet-based technology, mobile diagnostic imaging apps, and the development of a mobile tool for workflow optimization in academic radiology departments [27,28,31]. Furthermore, interesting perspectives are detected, such as musculoskeletal ultrasonography education with radiology training [13], the development and integration of innovative mobile devices in medicine [14,20], AI integration in pediatric radiology [16], the development and use of mobile mammography units in breast cancer screening [17], applications in simulation training [18], the development of models (including standards and quality control) in low resource settings [20], and integration in telemedicine models [15]. An important boost in all areas was given by the COVID-19 epidemic, such as in the integration with telemedicine [15]. In relation to telemedicine and the impact of COVID-19, this overview highlights how the intersection between mobile and domiciliary radiology has undergone significant transformations, driven by technological innovations and a growing emphasis on patient-centered care. A synthesis of various research themes sheds light on key trends and breakthroughs, providing a comprehensive understanding of the current landscape.

#### 3.2.1. Musculoskeletal Ultrasound

The exploration of musculoskeletal ultrasound as a radiation-free and dynamic imaging tool is a focal point in Neubauer et al.’s research [13]. This modality not only enhances diagnostic and therapeutic safety but also addresses the increasing demand for training opportunities. The recognition of mobile devices’ important role underscores the potential for standardized musculoskeletal ultrasound curricula to drive advancements in training and program implementation.

#### 3.2.2. Mobile Devices Integration in the Health Domain

Kufel et al. [14] delve into the challenges surrounding the widespread adoption of mobile devices in medicine. The study compares the usability of mobile applications for diagnostic image evaluation with stationary stations, highlighting both positive and negative features. The call for a universal mobile application underscores the need for seamless integration into hospital infrastructure, fostering future research on advancements in mobile devices in the medical sector.

#### 3.2.3. Telemedicine and COVID-19 Impact

Perrone et al.’s study [15] explores the transformative impact of the COVID-19 pandemic on healthcare organizations, emphasizing the evolution of telemedicine areas. Electronic health records, teleradiology, and mobile radiology emerge as crucial components, with telemedicine seen as complementary to traditional medicine, especially in radiology and dermatology applications.

#### 3.2.4. AI Applications in Pediatric Radiology

Addressing the critical aspect of radiation dose optimization in pediatric radiology, Ng et al. [16] focus on the dominance of deep convolutional neural networks in reducing radiation dose without compromising diagnostic information. The inclusion of mobile radiography devices in the study emphasizes the necessity for further exploration of AI’s value in optimizing doses for various modalities in pediatric radiology.

#### 3.2.5. Mobile Mammography for Breast Cancer Screening

Trivedi et al. [17] highlight disparities in breast cancer outcomes among different populations and stress the role of mobile mammography units in providing convenient screening services. The study underscores the potential of mobile mammography to enable earlier detection, particularly in underserved women, and to mitigate the impact of missed screenings during the COVID-19 pandemic.

#### 3.2.6. Simulation-Based Training (SBT)

Conducting a systematic review, Heuer et al. [18] identify the common use of simulation-based training in allied health professions. While SBT proves effective in enhancing short-term measures, its impact on sustained skill enhancement and patient outcomes remains unclear, suggesting a need for further investigation.

#### 3.2.7. Focus on Diagnostic Radiology Standards in Low-Resource Settings

Dinar et al. [20] focus on diagnostic radiology standards in low-resource settings, emphasizing the critical role of diagnostic imaging in crisis situations. The lack of evidence on standard quality control for mobile health units (MHUs) calls for investigations to assess the feasibility of different quality control standards in MHUs.

#### 3.2.8. Augmented Reality in Anatomy Education

McBain et al. [21] identify and assess various AR modalities used in anatomy education, emphasizing the need for sufficiently powered studies using validated assessment tools. The exploration of AR’s role in anatomical education extends to considerations of usability, feasibility, and acceptability.

#### 3.2.9. Domiciliary Radiology with Mobile X-ray Equipment

Toppemberg et al.’s [23] scoping review on domiciliary radiology explores the use of mobile X-ray equipment outside the hospital. While suggesting potential benefits for various populations, the authors emphasize the need for further research, noting limitations in language, databases, and grey literature.

#### 3.2.10. Trends in Ultrasound Imaging

Wang et al. [24] provide a comprehensive review of current trends in ultrasound imaging. The emphasis on widespread use, portability of mobile devices, and recent advancements, including developments in miniaturization and improved imaging performance, point towards a promising future for ultrasound in addressing various medical challenges.

#### 3.2.11. Quality Improvement Interventions in Radiology

Jabin et al. [26] review quality improvement interventions in radiology, showcasing improvements in operational efficiency, reported turnaround time, teamwork, communication, and patient safety. The positive outcomes associated with the introduction of mobile radiography underscore its potential to enhance overall workflow efficiency.

#### 3.2.12. Integration of Internet-Based Technology in Radiology

Gupta et al. [27] discuss the integral connection between radiology and the Internet, emphasizing the rapid rise of Internet-based technology. The seamless integration of Internet-based applications, including mobile devices and wearables, highlights the transformative impact on radiology workflows, education, research, and patient engagement.

#### 3.2.13. Mobile Diagnostic Imaging Device

Adusumilli et al. [28] review the Mobile MIM diagnostic imaging device, emphasizing its features and usability on iOS mobile devices. The focus on radiation treatment plans and medical image analysis highlights the potential of mobile applications in enhancing specific aspects of diagnostic imaging.

#### 3.2.14. Workflow Optimization with Mobile Tools

Makary et al. [31] address the challenges of communication and workflow efficiency in radiology operations. The development of a mobile tool to optimize workflow efficiency in academic radiology departments underscores the potential for mobile technologies to enhance communication, provide access to guidelines, and keep users informed.

In conclusion, this comprehensive overview of recent research in medical imaging demonstrates a rich tapestry of advancements, challenges, and potential avenues for future exploration. From musculoskeletal ultrasound to AI applications, telemedicine, and mobile technologies, the collective findings underscore the dynamic evolution of medical imaging and its pivotal role in advancing healthcare. As technology continues to shape the landscape, the intersection of innovation and patient-centered care remains at the forefront of the field’s progression. Table 1 reports the focus and the key points emerging for each study.

## 4. Discussion

The discussion is organized into five sections, each carefully rendered into distinct paragraphs. The *first opening paragraph* establishes the context by exploring the prevailing trends in the dissemination of knowledge within this domain. The subsequent *second paragraph* then unfolds in two facets: (I) a meticulous examination of the pivotal findings arising from the study results, with a keen focus on discerning emerging opportunities, and (II) an analysis of the limitations and areas demanding a more extensive investigation, aiming to provide a comprehensive perspective. The *third paragraph* discusses the key issue of *mobile radiology as a tool taking services directly to patients*, also integrating the overview with comparisons. The *fourth paragraph* provides the takeaway and emerging considerations. The *fifth and last paragraph* discusses the limitations of the study.

### 4.1. Numerical Trends

**Figure 1:**
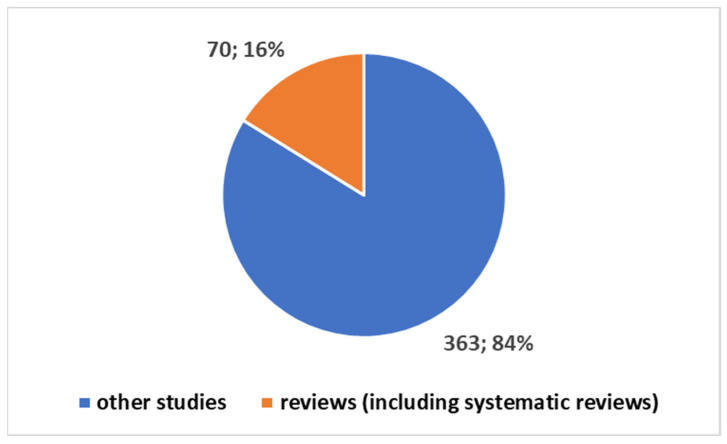
Trends in the studies since 1960.

**Figure 2:**
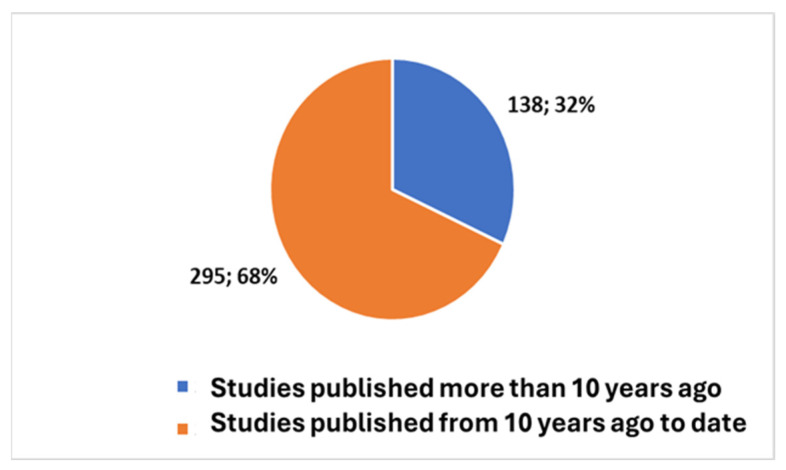
Trends in the studies with reference to the last ten years.

Exploring the evolving landscape of scientific publications in this domain using the first composite keywords in Box 1 yields valuable insights. We applied the research on the PubMed platform [32]. The initial references to these technologies date back to 1947 [1], marking the first time they came under discussion. However, it was not until 1958 that their mention gained prominence, particularly in the context of their applications within communities [1]. The cumulative count of publications since 1947 has surged to 433 [10], as depicted in Figure 1. Notably, 16% of these publications, totalling 70, are reviews (including systematic reviews). If we focus on the last decade (Figure 2), we observe that 68% of the studies have been produced during this period.

**Figure 3:**
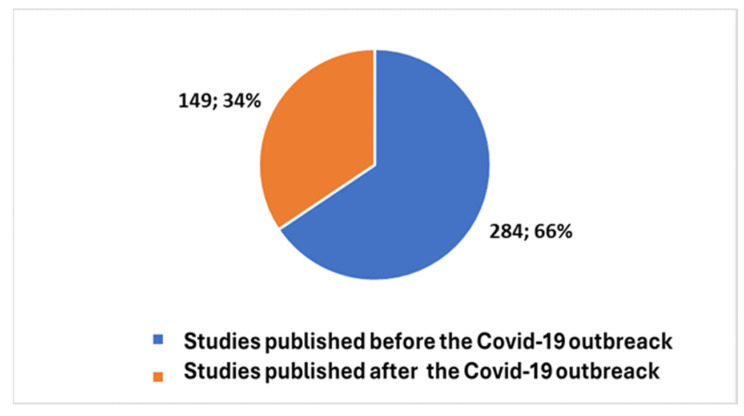
Trends of studies before and after the COVID-19 pandemic.

Upon scrutinizing the body of work generated in response to the COVID-19 pandemic to date, we uncovered a total of 149 studies, constituting a noteworthy 34% more than 1/3 of the total published papers. This revelation underscores that nearly one-third (as illustrated in Figure 3) of all studies conducted since 1947 have remarkably converged within the last four years. This trend serves as a compelling indication of how COVID-19 has not only accelerated advancements in this domain but has also acted as a driving force in various sectors linked to the integration of digital technologies within the patient’s domicile. This shift is particularly pronounced in efforts aimed at safeguarding the well-being of individuals who are frail or living with disabilities.

Box 1 **The proposed composite keys.**

*((“mobile”[All Fields] OR “mobiles”[All Fields]) AND “radiology”[Title/Abstract]) OR “mobile radiography”[Title/Abstract] OR ((“domiciliaries”[All Fields] OR “domiciliary”[All Fields]) AND “radiology”[Title/Abstract]) OR “domiciliary radiography”[Title/Abstract]*

*(Home radiology[Title/Abstract]) OR (Home radiography [Title/Abstract]) OR (Remote radiography[Title/Abstract]) OR (remote radiology[Title/Abstract]) OR (remote radiography[Title/Abstract])*

### 4.2. Interpretation of Results: Opportunities, Limitations, and Suggestions for a Broader Investigation

The explored reviews delve into a myriad of captivating realms within mobile/domiciliary radiology, encompassing a rich tapestry of subjects. These include the dynamic incorporation of augmented reality (AR) in anatomy education [21], the intricate domain of domiciliary radiology utilizing mobile X-ray equipment [23], cutting-edge advancements in ultrasound imaging [24], transformative quality improvement interventions [26], the seamless integration of Internet-based technology, mobile diagnostic imaging applications, and the development of a mobile tool for workflow optimization in academic radiology departments [27,28,31]. Amidst this vast landscape, intriguing perspectives surface, demanding focused attention on opportunities, limitations, and the identification of areas warrant a more comprehensive investigation. Noteworthy findings include the profound impact of musculoskeletal ultrasonography education on radiology training [13], the dynamic evolution of innovative mobile devices in medicine [14,20], the integration of Artificial Intelligence (AI) in pediatric settings [16], the pioneering development and utilization of mobile mammography units for breast cancer screening [17], the revolutionary applications in simulation training [18], the imperative need for model development (including standards and quality control) in resource-limited settings [20], and the integration of these technologies into evolving telemedicine models [15].

The catalyzing force of the COVID-19 epidemic has served as a transformative catalyst across all these domains, which is especially evident in the enhanced integration with telemedicine [15]. This pivotal moment underscores the urgency of not only acknowledging but also meticulously examining the opportunities, limitations, and potential avenues for broader investigation that have unfurled within the intricate intersections of mobile/domiciliary radiology and the evolving healthcare landscape.

#### 4.2.1. Emerging Opportunities

In the rapidly evolving landscape of the investigated field, a tapestry of emerging opportunities unfolds, promising to reshape the way we approach diagnostics, training, and patient care. Technological advancements stand at the forefront, offering a dual promise of precision and accessibility.

One notable opportunity lies in the realm of musculoskeletal ultrasound education, as illuminated by Neubauer et al. [13]. The emphasis on a radiation-free and dynamic imaging tool not only enhances diagnostic and therapeutic safety but also addresses the escalating demand for training. The recognition of mobile devices as pivotal in this landscape underscores the potential for standardized curricula, ushering in a new era of proficiency and program implementation. In tandem, the integration of mobile devices in medicine, as explored by Kufel et al. [14], presents a dynamic frontier. The challenges associated with widespread adoption become a call to action, emphasizing the need for a universal mobile application seamlessly integrated into the hospital infrastructure. This not only addresses current hurdles but also paves the way for future advancements in the utilization of mobile devices within the medical sector. The transformative impact of the COVID-19 pandemic has opened vistas for the evolution of telemedicine, as uncovered by Perrone et al. [15]. Electronic health records, teleradiology, and mobile radiology emerge as key players, synergizing with traditional medicine, particularly in the domains of radiology and dermatology. The pandemic, while challenging, acted as a catalyst for telemedicine’s complementary role. AI took center stage in pediatric radiology, as elucidated by Ng et al. [16]. The dominance of deep convolutional neural networks in reducing radiation dose without compromising diagnostic information heralds a new era. The study’s inclusion of mobile radiography devices underlines the necessity for further exploration, suggesting AI’s pivotal role in optimizing doses across diverse modalities.

Breast cancer screening, a perennial concern, finds an ally in mobile mammography units, as highlighted by Trivedi et al. [17]. The disparities in outcomes between different populations become a focal point, and mobile mammography emerges as a solution, particularly for underserved women. Its potential to enable earlier detection and mitigate the impact of missed screenings, especially in the wake of the COVID-19 pandemic, marks a significant stride forward. Simulation-based training (SBT) in allied health professions, per Heuer et al. [18], presents a dual narrative. While proving effective in enhancing short-term measures, the sustained impact on skill enhancement and patient outcomes remains elusive. This ambiguity becomes a clarion call for further investigations, opening the door for refining training methodologies. The global discourse expands to encompass diagnostic radiology standards in low-resource settings, as investigated by Dinar et al. [20]. The emphasis on the critical role of diagnostic imaging in crisis situations unveils an urgent need for standard quality control for mobile health units (MHUs). This beckons investigations to assess the feasibility of different quality control standards, laying the groundwork for successful patient management in challenging environments. AR in anatomy education, as explored by McBain et al. [21], offers a visionary perspective. The identification and assessment of AR modalities lay the groundwork for an immersive educational experience. The call for sufficiently powered studies becomes a clarion call, offering the potential for a paradigm shift in anatomical education methodologies. Domiciliary radiology, with a focus on mobile X-ray equipment, unfolds as a promising frontier, as revealed by Toppemberg et al. [23]. While potential benefits for diverse populations surface, the need for further research becomes apparent. This highlights the nascent nature of this avenue, urging researchers to delve deeper into language, databases, and grey literature.

The discourse broadens to encompass trends in ultrasound imaging, a domain meticulously reviewed by Wang et al. [24]. The emphasis on widespread use, portability of mobile devices, and recent advancements becomes a harbinger of progress. Developments in miniaturization and improved imaging performance set the stage for ultrasound’s promising future, extending its applications into molecular imaging and focused treatments. Quality improvement interventions in radiology, as reviewed by Jabin et al. [26], form a nexus of positive outcomes. The integration of mobile radiography demonstrates improvements in operational efficiency, report turnaround time, and teamwork. The call for further research becomes an invitation to explore additional dimensions of quality, cost, and risk versus benefit, sculpting a roadmap for enhanced outcomes. The integral connection between radiology and the Internet, as discussed by Gupta et al. [27], opens up a new frontier. The seamless integration of Internet-based applications, including mobile devices and wearables, sets the stage for transformative changes. The impact of the Internet of Things (IoT) on radiology workflows, education, research, and patient engagement becomes a realm ripe for exploration. The discourse turns towards a review of the Mobile MIM diagnostic imaging device by Adusumilli et al. [28]. The emphasis on features and usability, particularly on iOS mobile devices, heralds a niche in enhancing specific aspects of diagnostic imaging. The focus on radiation treatment plans and medical image analysis uncovers a realm where mobile applications can become indispensable tools. Challenges in communication and workflow efficiency within radiology operations find a solution in the development of mobile tools, as shared by Makary et al. [31]. The optimization of workflow efficiency in academic radiology departments becomes a testament to the transformative power of mobile technologies. The tool’s development, aimed at enhancing communication, providing access to guidelines, and keeping users informed, marks a pivotal moment in radiology operations.

#### 4.2.2. Emerging Limitations and Suggestions for a Broader Investigation

From the analysis of the reviews, important limitations emerge, and indirectly, suggestions for a broader investigation are included in Table 2.

Musculoskeletal ultrasound education, as illuminated by Neubauer et al. [13], grapples with its own set of limitations. Despite its potential as a radiation-free and dynamic imaging tool, the challenge lies in developing standardized curricula. The diversity in course concepts and programs across disciplines, coupled with the emphasis on mobile devices, highlights the need for careful consideration of educational frameworks that cater to the unique demands of different specialties. The integration of mobile devices in medicine, as explored by Kufel et al. [14], faces hurdles in widespread adoption. Usability comparisons between mobile applications and stationary stations reveal a dichotomy in procedures, device availability, and regulatory frameworks. The envisioned universal mobile application, while promising, must contend with the intricate nuances of hospital infrastructure, calling for meticulous development and integration strategies.

The transformative impact of the COVID-19 pandemic on telemedicine, as discussed by Perrone et al. [15], also unravels limitations. While electronic health records, teleradiology, and telemedicine applications emerged as crucial during the pandemic, challenges in fully replacing traditional medicine persist. The integration of telemedicine into certain specialties, particularly radiology and dermatology, requires a delicate balance to ensure comprehensive patient care. AI optimization in pediatric radiology, per Ng et al. [16], encounters limitations despite its promise in dose optimization. The dominance of deep convolutional neural networks is not without challenges, and the study emphasizes the necessity for further exploration. The nuanced considerations of AI’s value in optimizing doses for various modalities in pediatric radiology underscore the intricate balance needed to ensure both diagnostic accuracy and radiation safety. Mobile mammography for breast cancer screening, as explored by Trivedi et al. [17], grapples with challenges in addressing disparities. While mobile mammography units hold promise, reaching underserved populations and mitigating the impact of missed screenings during the COVID-19 pandemic is no small feat. The effectiveness of these units hinges on overcoming logistical, financial, and infrastructural barriers. Simulation-based training (SBT) in allied health professions, per Heuer et al. [18], demonstrates effectiveness in short-term measures but reveals uncertainties in long-term impacts. The sustained enhancement of skills and its translation into improved patient outcomes remain elusive, necessitating further investigations to decipher the nuanced dynamics of SBT. Diagnostic radiology standards in low-resource settings, according to Dinar et al. [20], confront the challenge of a lack of evidence on standard quality control for mobile health units (MHUs). The call for investigations to assess the feasibility of different quality control standards in MHUs highlights the inherent complexities in maintaining diagnostic imaging standards in crisis situations. AR, in anatomy education, as explored by McBain et al. [21], grapples with the need for sufficiently powered studies. While AR modalities hold promise in enhancing anatomical education, the current discourse stresses the importance of validated assessment tools and comprehensive studies to understand the full scope of AR’s impact. Domiciliary radiology with mobile X-ray equipment, as outlined by Toppemberg et al. [23], unveils limitations in language, databases, and grey literature. The potential benefits suggested by the studies need to be interpreted within the confines of these limitations, calling for a cautious approach to generalize findings and a comprehensive exploration of the existing gaps. Future trends in ultrasound imaging, as reviewed by Wang et al. [24], encounter limitations in the breadth of their projections. While the emphasis on widespread use, portability of mobile devices, and recent advancements paints a promising picture, the feasibility and applicability of these trends in diverse healthcare settings warrant careful consideration. Quality improvement interventions in radiology, per Jabin et al. [26], underscore the need for further research to explore additional dimensions of quality, cost, and risk versus benefit. While the introduction of mobile radiography demonstrates improvements in various aspects, a nuanced understanding of its limitations is crucial to refine and optimize its integration into radiology workflows. The integral connection between radiology and the Internet, as discussed by Gupta et al. [27], faces challenges in the rapid rise of Internet-based technology. The transformative impact of the Internet of Things (IoT) on radiology workflows, education, research, and patient engagement necessitates a thorough understanding of the potential risks and ethical considerations associated with such integration. The review of the Mobile MIM diagnostic imaging device by Adusumilli et al. [28] brings to light limitations in compatibility, particularly with mammography. The strengths and limitations of the app, while notable, call for a nuanced approach to its usage, acknowledging its role while considering alternative solutions for specific diagnostic needs. Addressing challenges in communication and workflow efficiency within radiology operations, as shared by Makary et al. [31], while optimizing workflow efficiency with mobile tools underscores the need for comprehensive solutions. While mobile tools show promise in enhancing communication and providing access to guidelines, their broader integration into complex healthcare systems demands meticulous planning and continuous refinement.

### 4.3. Mobile Radiology: Taking Services Directly to Patients

The dynamic interplay between mobile radiology and domiciliary radiology, explored through insightful reviews, unveils nuanced themes and intricacies, shedding light on both emerging opportunities and challenges within this intersection. This vibrant convergence exposes a kaleidoscope of diverse interests, yet the true amalgamation dedicated to delivering radiology services to patients at home or in alternative locations like protected residences or service dispensing kiosks has received comparatively limited attention in existing studies. Amidst this landscape, a solitary review study identified in our research [23] delves into this specific facet, prompting a call for a deeper exploration to address the identified limitations. Our comprehensive overview underscores a significant gap: despite the inception of domiciliary radiology as a discipline in 1958 [1], there remains a conspicuous dearth of substantial reviews in this domain. As a consequence, a shift toward recent, specific studies (not only reviews overviewed by us), not captured by our search keywords, becomes imperative, facilitating more in-depth comparative analyses. In an effort to delve further into this targeted exploration, the discourse on domiciliary radiology unfolds as a narrative spanning decades. This narrative not only captures historical landmarks but also integrates contemporary research, providing a comprehensive understanding of the evolutionary trajectory of domiciliary radiology. This multifaceted approach allows for a more nuanced exploration of the discipline, paving the way for a deeper comprehension of its complexities, challenges, and potential avenues for advancement in the realm of healthcare delivery. Originating in 1958, Losev’s pioneering work aimed at enhancing roentgenological services in villages through improved mobile X-ray units sets the stage for subsequent developments [1]. Examining General Practitioners’ attitudes toward domiciliary radiography in 1995, Sawyer et al.’s survey [2] unveils the perceived significance of the service. The findings indicate a consensus among practitioners regarding its importance, with potential implications highlighted if the service were to be discontinued. However, the nuanced variations in opinions on different examinations underscore the intricate challenges associated with integrating domiciliary radiology into routine healthcare practices [2]. In recent times, Mark et al.’s (2022) [3] establishment of a domiciliary-based X-ray response team marks a practical advancement. This initiative not only garnered positive feedback from patients but also demonstrated a substantial reduction in avoidable conveyance, offering a promising avenue for improving patient care and experiences. While advancements in domiciliary radiology showcase promising outcomes, the research by Toppenberg et al. (2020) [6] in the form of a randomized controlled trial reveals the inherent complexities involved. Challenges such as doctor withdrawal and randomization issues underscore the need for careful consideration in designing and executing studies within this domain. Operational challenges are brought to light by Andersen et al. (2023) [33], shedding insights into the intricacies of setting up a mobile X-ray unit. This study emphasizes the dual impact of increased physicality for radiographers and positive outcomes for vulnerable patients who can undergo examinations in familiar surroundings. Exploring the patient’s perspective, Dollard et al. (2022) [34] delve into residents’ views on mobile X-ray services in aged care facilities. The positive reception among residents, coupled with the emphasis on equivalent quality services in familiar surroundings, reinforces the patient-centric nature of domiciliary radiology [34]. In economic terms, Kjelle et al.’s (2019) [35] cost analysis showcases a 30% reduction in costs with the implementation of mobile radiography services for nursing home residents. Aldridge et al.’s study [36] assesses the impact of volunteer peer educators on the uptake of mobile X-ray tuberculosis screening at homeless hostels. Conducted as a cluster randomized controlled trial in London, the research involved 46 hostels and 2342 residents. Despite the intervention’s meticulous design, results revealed no substantial evidence that volunteer peer educators significantly increased screening uptake. The study emphasizes the need for further qualitative investigations to explore potential ancillary benefits associated with the involvement of peer volunteers. In essence, the research underscores the challenges in leveraging peer educators to enhance tuberculosis screening participation in homeless populations.

This economic viability underscores the potential for domiciliary radiology to not only enhance patient care but also offer a pragmatic solution in terms of cost-effectiveness. Kjelle and Lysdahl (2017) [37] consolidate evidence on the benefits of mobile radiography services, emphasizing reduced hospital transfers and timely diagnosis. This broad analysis reaffirms the potential advantages that domiciliary radiology can bring to the healthcare landscape. In a broader societal context, public–private partnerships, as exemplified by Datta et al. (2017) [38], illustrate the potential impact of collaborative efforts in addressing healthcare gaps. The success of this specific initiative in detecting pulmonary TB highlights the broader role such partnerships can play in scaling up and designing impactful interventions.

It is also crucial to take into account that the realm of radiology is currently undergoing profound transformations driven by technological innovations, and these advancements shed light on crucial considerations that demand careful attention, particularly when discussing domiciliary radiology. It becomes imperative to factor in both the existing social disparities and the emerging technological potentials. Geographic and ethnic variations can introduce significant inequalities in access to the healthcare system, especially in the realm of radiological services [39]. Hence, a thorough examination is necessary to deliberate and implement solutions that not only acknowledge but actively work toward enhancing inclusivity.

Moreover, a comprehensive evaluation of solutions is required to facilitate broader access to emerging opportunities facilitated by technological innovations. Cloud technology, for instance, is increasingly playing a pivotal role in radiology, making substantial contributions to both environmental and economic aspects [40]. Decision-makers in the field of domiciliary radiology must conduct a meticulous analysis, weighing the benefits against the costs to make informed decisions that harmonize with both economic and environmental sustainability.

In addition, the novel imaging solutions brought forth by cutting-edge technologies, encompassing advancements in imaging techniques and detectors [41,42], ought to be intricately considered by all stakeholders involved in domiciliary radiology services. This is crucial given the transformative potential these innovations hold and the opportunities they can offer in advancing the landscape of domiciliary radiology.

In addition, there are specific challenges in domiciliary radiology, particularly given that digital healthcare is brought into homes along with the necessary equipment. This introduces new paradigms. Therefore, studies should also address potential challenges associated with the logistics of transporting radiological equipment to patients’ homes. This endeavor involves more than only technical considerations; it should encompass the need for infrastructural support, such as the presence of elevators and the availability of dedicated technicians. Furthermore, factors related to the application context, such as the complexities of providing services in large and congested cities, including issues of timely service delivery due to traffic, parking difficulties, and other logistical constraints, should be carefully addressed. Additionally, a thorough analysis is needed regarding the requirements for an appropriate reporting workstation. Essential questions arise, such as determining the minimum characteristics necessary for accurate reporting and identifying the type of screen required. This becomes especially crucial when dealing with mammography reporting, where precision is paramount. Another critical aspect to consider is the communication of the report to patients, given that they may not have easy access to hospitals. All these aspects could be the subject of targeted studies, also based on surveys involving relevant stakeholders. Surveys are indeed valuable for collecting experiences, constructive feedback, identifying challenges, and proposing solutions. Such surveys could be conducted through convenient tools like Computer-Aided Web Interviewing, as seen in other experiences [43].

*Currently, a rapid overview of recent surveys in teleradiology, apart from not focusing on domiciliary radiology, does not highlight a focus on these strategic issues*.

The topics covered in these studies include: the examinations of teleradiology practices in Turkey [44], the perceptions of clinical medical students towards radiology careers in Ghana [45], patient-reported outcomes after fracture treatment in primary healthcare [46], usability and efficiency evaluations of an application in orthopedics [47], the socio-economic and psychological impacts of the COVID-19 outbreak on radiologists [48], work-style reform and technology utilization among diagnostic radiologists in Japan [49], skepticism about artificial intelligence in the radiology field [50], patient satisfaction with teleradiology services in Italy [51], patient satisfaction with teleradiology services in general practice [52], on-call service of neurosurgeons in Germany [53], attitudes of Korean primary care family physicians toward telehealth [54], Factors influencing clinician satisfaction with radiology services [55], and positive aspects found in healthcare information and communication technology implementation in Finland [56].

*In light of the absence of the above-mentioned themes in these studies* [44,45,46,47,48,49,50,51,52,53,54,55,56], *it is recommended that researchers address these issues promptly*.

Overall, the narrative on domiciliary radiology, weaving through historical foundations, practitioner and patient perspectives, operational challenges, economic considerations, and collaborative models, paints a vivid picture of an evolving field. As we navigate these intricacies, the synthesis of these facets not only guides future research and implementation strategies but also underscores the potential of domiciliary radiology in transforming healthcare delivery.

Collectively, these studies underscore the imperative need for a comprehensive approach to technology assessment that can effectively navigate diverse domains. Foremost among these domains is the technological realm, where advancements and innovations play a pivotal role. Equally critical is the economic domain, where a thorough cost–benefit analysis becomes a linchpin for informed decision-making.

Of particular interest is the identification of application domains, extending the impact of technology assessment from fragile patient populations to those experiencing homelessness. This inclusive approach speaks to the broader societal implications and potential benefits that can be derived from a well-executed technology assessment strategy.

Noteworthy is the emphasis on monitoring, a dynamic process encompassing both social aspects and general outcomes. The integration of targeted surveys adds depth to the evaluative process, offering insights into the nuanced intersections of technology with human experiences.

A salient recommendation emanating clearly from these studies is the strategic focus on multi-domain technology assessment initiatives. The endorsement and sponsorship of such initiatives by scientific societies could serve as a catalyst for their effectiveness. Additionally, in the realm of monitoring tools, the utilization of computer-aided web interviewing tools [43] emerges as a valuable resource, contributing to the robust evaluation of technological interventions.

Within the ambit of technology assessment, regulatory considerations assume paramount importance. This extends to encompassing the regulatory landscape for both Medical Devices and radiation protection, addressing critical aspects that ensure the responsible and ethical integration of technology into healthcare practices.

In essence, these studies advocate for a holistic and interdisciplinary approach to technology assessment, urging the inclusion of regulatory frameworks and endorsing collaborative efforts supported by scientific societies. The landscape of technology assessment should not only be innovative but also ethical, ensuring that technological advancements align with societal needs and well-defined regulatory standards.

### 4.4. Final Takeaway Message

In navigating mobile and domiciliary radiology research, a key takeaway emerges: ongoing advancements, including AI integration and telemedicine applications, present opportunities for reshaping healthcare diagnostics. However, challenges exist, emphasizing the need for a vigilant recognition of the limitations. To propel the field forward, a dedicated focus on multi-domain technology assessment is crucial, especially when providing mobile radiology at patients’ homes or point-of-care settings. This involves assessing technological, economic, social, and regulatory aspects for a comprehensive understanding. Through conscientious exploration and commitment to precision, we unlock the true potential of mobile/domiciliary radiology, fostering advancements across healthcare.

### 4.5. Limitations

The study conducted a comprehensive analysis by scrutinizing two prominent databases, PubMed and Scopus. It deliberately excluded other databases primarily concentrated on technological advancements, irrespective of their medical implications. This deliberate choice in database selection was made with the specific aim of honing in on a thorough examination of both the technological developments and their direct applicability to the medical domain. In doing so, the study aimed to provide a nuanced and detailed insight into the current state of the art at the intersection of technology and medicine.

## 5. Conclusions

In conclusion, the overview of mobile and domiciliary radiology, derived from a comprehensive study review, reveals an intriguing and promising outlook. Noteworthy advancements in this dynamic field include the integration of AI, groundbreaking applications in telemedicine, and the educational potential of mobile devices. The post-COVID-19 landscape witnesses significant progress in telemedicine evolution and the impactful role of AI in pediatric radiology. Mobile mammography units emerge as a solution for underserved women, underscoring the critical importance of early detection in breast cancer screening. Exploring domiciliary radiology, particularly with mobile X-ray equipment, signals a promising frontier, urging in-depth research for comprehensive insights into its potential benefits for diverse populations. Limitations have been identified, along with suggestions for future exploration. Various domains within mobile and domiciliary radiology, encompassing musculoskeletal ultrasound education, mobile device integration in medicine, telemedicine, AI applications in pediatric radiology, and mobile mammography for breast cancer screening, present specific challenges. Addressing these challenges requires thorough investigations and tailored approaches. Additional areas like simulation-based training, diagnostic radiology standards in low-resource settings, augmented reality in anatomy education, domiciliary radiology with mobile X-ray equipment, trends in ultrasound imaging, quality improvement interventions, Internet-based technology integration, mobile diagnostic imaging devices, and workflow optimization with mobile tools each pose unique considerations, emphasizing the need for extensive research efforts and specialized approaches in their respective domains. A crucial recommendation underscores the importance of strategically prioritizing multi-domain technology assessment initiatives, with the endorsement and sponsorship of scientific societies playing a pivotal role. Furthermore, regulatory considerations, an integral facet of technology assessment, are emphasized as paramount. These considerations span both Medical Devices and radiation protection, aiming to guarantee responsible and ethical technology integration in healthcare practices. The broader landscape of technology assessment should strive to be innovative, ethical, and in alignment with societal needs and regulatory standards.

## Figures and Tables

**Table 2:** Focus and suggestions for a broader investigatio.

Focus [Reference ID]	Suggestion
*Musculoskeletal Ultrasound* *Education* [13]	Undertake a comprehensive, cross-disciplinary investigation to understand the nuanced training needs in musculoskeletal ultrasound across diverse medical specialties. This broader exploration could inform the development of educational frameworks tailored to the specific requirements of each field.
*Mobile Devices Integration* *in Medicine* [14]	Engage in an interdisciplinary examination of the impediments to the widespread adoption of mobile devices in medical settings. Broader investigations involving the collaboration between healthcare professionals, technology experts, and policymakers can pave the way for comprehensive guidelines for seamless integration.
*Telemedicine and* *COVID-19 Impact* [15]	Initiate longitudinal studies to assess the enduring impact of telemedicine on patient outcomes, healthcare costs, and the quality of physician-patient interactions. A broader investigation can delve into the integration of telemedicine into routine medical practices, ensuring its sustainability and effectiveness beyond crisis situations.
*AI Applications in Pediatric* *Radiology* [16]	Expand the scope of AI applications in pediatric radiology by exploring the intricacies of different imaging modalities and clinical scenarios. Collaborate with pediatric specialists to tailor AI algorithms to diverse patient populations and medical conditions.
*Mobile Mammography* *for Breast Cancer Screening* [17]	Conduct in-depth studies to identify and address logistical, financial, and infrastructural barriers in implementing mobile mammography units. Broader investigations could involve partnerships with community organizations to develop targeted strategies for reaching underserved populations.
*Simulation-Based Training (SBT) in Allied Health Professions* [18]	Undertake longitudinal studies to assess the sustained impact of simulation-based training on skill enhancement and patient outcomes. Broader investigations might involve exploring innovative approaches within SBT, such as incorporating virtual reality or gamification elements.
*Diagnostic Radiology Standards in Low-Resource Settings* [20]	Collaborate with international organizations and healthcare providers to establish evidence-based standards for quality control in mobile health units (MHUs) in low-resource settings. Broader investigations could involve assessing the feasibility of different quality control standards and adapting them to the specific challenges of crisis situations.
*Augmented Reality in Anatomy Education* [21]	Extend the scope of AR applications in anatomy education by exploring their effectiveness in diverse educational settings. Broader investigations might involve collaborations with educational institutions to implement AR-enhanced curricula and assess their impact on student learning outcomes.
*Domiciliary Radiology with Mobile X-ray Equipment* [23]	Collaborate with diverse healthcare providers and communities to identify potential benefits and challenges in the use of mobile X-ray equipment outside the hospital. Broader investigations could contribute to optimizing the implementation of such technology for various populations.
*Trends in Ultrasound Imaging* [24]	Explore evolving trends in ultrasound imaging, emphasizing not only technological advancements but also the societal and economic factors influencing its widespread use. Broader investigations could shed light on the transformative potential of ultrasound in addressing broader healthcare challenges.
*Quality Improvement Interventions in Radiology* [26]	Investigate additional dimensions of quality improvement interventions in radiology, including cost-effectiveness and a comprehensive risk versus benefit analysis. Broader investigations could contribute to a more holistic understanding of the impact of interventions on various aspects of healthcare delivery.
*Integration of Internet-Based Technology in Radiology* [27]	Delve into the evolving landscape of Internet-based technology in radiology, with a focus on its impact on medical education and patient engagement. Broader investigations could explore the intersection of technology, education, and patient care, shaping the future of radiology workflows.
*Mobile Diagnostic Imaging Device* [28]	Explore the potential applications of mobile diagnostic imaging devices in diverse medical scenarios. Broader investigations could focus on expanding the functionalities and compatibility of such devices, addressing current limitations and enhancing their overall utility.
*Workflow Optimization with Mobile Tools* [31]	Investigate the broader implications of workflow optimization with mobile tools in radiology operations. Broader investigations could explore innovative approaches to enhance communication, streamline access to information, and improve overall efficiency in academic radiology departments.

## Data Availability

Not applicable.
